# Ecosystem regime change inferred from the distribution of trace metals in Lake Erie sediments

**DOI:** 10.1038/srep07265

**Published:** 2014-12-01

**Authors:** Fasong Yuan, Richard Depew, Cheryl Soltis-Muth

**Affiliations:** 1 Department of Biological, Geological, and Environmental Sciences, Cleveland State University, 2121 Euclid Avenue, Cleveland, OH 44115, USA; 2 Northeast Ohio Regional Sewer District, 3900 Euclid Avenue, Cleveland, OH 44115, USA

## Abstract

Many freshwater and coastal marine ecosystems across the world may have undergone an ecosystem regime change due to a combination of rising anthropogenic disturbances and regional climate change. Such a change in aquatic ecosystems is commonly seen as shifts in algal species. But considerably less detail is known about the eutrophication history in terms of changes in algal productivity, particularly for a large lake with a great deal of spatial variability. Here we present an analysis of trace metals (Cu, Ni, Cd, and Pb) on a sediment core recovered from Lake Erie, off the Vermilion coast of northern Ohio, USA, to reconstruct the eutrophication history of the lake over the past 210 years. Following a slow eutrophication during European settlement, Lake Erie experienced a period of accelerated eutrophication, leading to an ecosystem regime transition into a eutrophic lake state in 1950. Our results suggested that the lake's biological productivity has ever since maintained fairly high even though a significant input reduction was realized from rigorous nutrient abatements that began as early as in 1969. This work underscored the role of in-lake biogeochemical cycling in nutrient dynamics of this already eutrophic lake.

Since European settlement in the early 1800s, Lake Erie has suffered from a range of natural and anthropogenic perturbations such as overfishing, toxic metal pollution, synthetic organic contamination, nutrient enrichment, exotic species invasions, and regional climate change^1^,^2^,^3^,^4^. Eutrophication of the lake is among the most serious problems^5^,^6^, posing an imminent threat to safe drinking water supply and ecosystem sustainability. Over the last several decades, much of the research has been directed to improve our understanding of the major mechanisms that led to the trophic changes in the lake. Although progress has been made in identifying the physical, chemical, and biological linkages, there is much uncertainty about the nature and causes of recent “re-eutrophication” in Lake Erie^6^,^7^,^8^. The relative importance of the in-lake biogeochemical cycling (as altered by exotic species invasions and climate change) is not firmly established^8^,^9^,^10^. There is a lack of observational data that can track multiple variables simultaneously to more accurately delineate the ecosystem changes^5^ that have occurred over the past two centuries. Existing phytoplankton biomass records from direct measurements of lake waters are rather sporadic (only available for limited project years at several sparsely-distributed locations) and fairly inconsistent^10^,^11^,^12^.

As an alternative to these costly conventional monitoring programs, analysis of sediment cores can provide a convenient multiproxy archive for evaluating long-term ecosystem changes that have occurred in a watershed^13^. Past work on Lake Erie has showed that deposits from certain locations have been well preserved and suited to recording ancient climatic and environmental conditions^14^,^15^,^16^. A unimodal pattern of rise and fall in sediment Pb concentrations has been documented to reflect the historical introduction and phase-out of leaded gasoline^16^,^17^,^18^,^19^,^20^. Attempts to reconstruct the eutrophication history of Lake Erie have also been made through analysis of carbon isotopic ratio (δ^13^C) of bulk organic and inorganic carbon in sediments^15^,^21^. These efforts revealed some important aspects on changes in metal pollution and lacustrine productivity in the lake. However, little is known about the nature and magnitude of the ecosystem changes in the lake.

Here we report an analysis of trace metals (Cu, Ni, Cd, and Pb) in sediments from Lake Erie to document a detailed history of the ecosystem changes over the past two centuries. We hypothesized that changes in the trophic lake state can affect biological productivity and subsequently alter the distribution and bioavailability of trace metals. These individual trace elements may behave differently in response to changes in the lake's trophic state and biological productivity. Thus, the distribution of trace metals in sediments can be used to reveal the trophic changes in a lake. Although the use of trace metals as paleoproductivity proxies has been well established for marine ecosystems^22^,^23^, their applicability and usefulness for reconstructing lacustrine paleoproductivity has not been explored sufficiently^24^. This is particularly the case for Lake Erie though multiple efforts were made to investigate the evolving history of anthropogenic metal inputs^16^,^17^,^19^,^20^.

The main goal of this work is to improve our understanding of the eutrophication history from the distribution of trace metals in a sediment core (V12) from the Sandusky basin in Lake Erie, about 65 km west of Cleveland in northern Ohio (Figure 1). The Sandusky basin was chosen for this investigation mainly because its position allows the accumulation of sediments that contain some trace metals transported from the most productive western basin upstream^25^,^26^. Sandwiched between the western and central Lake Erie basins, the Sandusky is a small, shallow, and cyclonic-flow-dominated basin^27^. The majority of the lake's algal blooms occur in the shallowest unstratified western basin, with a broad west-east flow regime driven by a large discharge (5,300 m^3^/s) from the Detroit River^27^. In contrast, the formation of late-summer hypoxia occurs mostly in the deeper stratified central basin^28^, featured with a large anticyclonic circulation^27^. Sediment samples from core V12 were measured on trace metals and dated to document a detailed history of the ecosystem changes in the Sandusky basin of Lake Erie (see Methods). We derived ratios of Cu/Ni, Pb/Cu, and Cd/Pb from our trace metal measurements and compared them with historical lake-level records and other previously published data from the central and eastern basins for a better understanding of basin-wise changes in the trophic state of the lake over the past 210 years.

## Results and discussion

### Trace metal concentrations

Profiles of Cu, Cd, Pb, and Ni concentrations in sediments from the Sandusky basin exhibit a broadly consistent pattern of variability (Figure 2). Their values remained near the background levels in the basal 10-cm section (prior to 1880), increased gradually until 1950, increased rapidly between 1950 and 1970, and subsequently decreased towards the sediment-water interface. The trace metal concentrations of the core-top sediments are comparable with those measured on surficial sediments in the western basin^29^,^30^ (Table 1). Our sediment chronology is in good agreement with previously published Pb records from the central (C94)^16^ and eastern (E91)^18^ basins (Figure 3). These records document a detailed history of Pb pollution: a minor increase from coal-burning between 1870 and 1910, a moderate increase from the introduction of leaded gasoline between 1910 and 1950, a large increase from effluents of industrial and municipal sources between1950 and1970, and a reduction from the phase-out of leaded gasoline begun in 1970^16^,^18^,^20^,^31^. Nevertheless, there are some distinctive features. For example, the rates of reduction in Pb as recorded in the Sandusky basin were not as fast as those revealed from the central and eastern basins (Figure 3). More importantly, our sediment record from the Sandusky basin exhibits some inter-metal variations. The reduction rates of Cu and Ni appeared to be to some extent slower than those of Cd and Pb (Figure 2).

### Enrichment factors

In fact, Ni, Cu, Pb, and Cd were enriched to varying degrees, for instance, in 1970 by factors of approximately 3, 4, 7, and 24, respectively. To evaluate the degree of ecosystem changes in the lake, we derived ratios of Cu/Ni, Pb/Cu, and Cd/Pb from our analytical results to reveal changes in the relative metal enrichment factors over the past two centuries (Figure 4). The 210-year record can be roughly divided into three periods based on trends and changes in these metal ratios and the historical changes in energy use^31^: European settlement before 1880, coal-dominated industrial (1880–1950), and petroleum-dominated industrial after 1950. First, the European settlement era (ESE) is characterized with low values of these metal ratios, a slow increase in Cu/Ni, and neither detectable nor consistent trends in Pb/Cu and Cd/Pb. Second, the coal-dominated industrial era (CIE) is marked by substantial increases in these metal ratios, indicating a large and long-lasting transition from a slow metal enrichment in 1880 to a fast metal enrichment in 1950. Lastly, during the petroleum-dominated industrial era (PIE), the three metal ratios behaved rather differently. Cu/Ni increased progressively, peaked in 1960, and increased again progressively. Cd/Pb continued to increase to high values (0.034) until 1960, and leveled off afterwards. Pb/Cu increased slightly in 1950s and rapidly in 1960s, peaked in 1970, and declined rapidly in 1990s. All these distinct features suggest that a substantial ecosystem change has occurred, which resulted in enhanced metal differentiation processes in the lake.

The most striking aspect of the 210-year metal record is the large and long-lasting increasing trends of trace metal ratios that suggest an ecosystem regime change occurred in the CIE. The Cu/Ni, Pb/Cu, and Cd/Pb ratios were naturally low, on average, 0.50, 0.70, and 0.010 during the ESE, and increased 68%, 51%, and 240% during this ecosystem transition (Table 1). Based on the early estimates of anthropogenic metal inputs into Lake Erie^32^, their trace metal ratios (1.27 for Cu/Ni, 2.10 for Pb/Cu, and 0.10 for Cd/Pb) were considerably larger than these background values. It is plausible to attribute the substantial increases in these trace metal ratios to enhanced contributions from anthropogenic sources. As discussed below, however, the changes in anthropogenic inputs alone are not sufficient to account for the variations of trace metal distributions in Lake Erie sediments. There are multiple lines of evidence indicating that the distributions of trace metals are significantly affected by changes in the trophic state and biological productivity of the lake^26^,^33^,^34^.

### Cu/Ni as a proxy of lacustrine productivity

We take the sedimentary Cu/Ni variations as a nearly direct measure of variations in Cu/Ni of suspended particulate matter to reflect changes in trace metal inputs, biological productivity, and other factors. In addition to local inputs from industrial and municipal effluents, most of the Cu and Ni in Lake Erie are derived from the upper Great Lakes via the Detroit River^26^. Although the overall quality of water and sediment from the Detroit River has improved since 1980s^35^, we argue that changes in Cu/Ni of the total anthropogenic inputs may have been minimal and that Cu/Ni of the lake can be indicative of changes in lacustrine productivity. Our Cu/Ni data suggest that the eutrophication of Lake Erie may have begun as early as in 1880, attributed to an enhanced nutrient loading from sediment erosion caused by forest clearance during European settlement^4^,^15^. The eutrophication accelerated remarkably during the CIE, in which a 0.3 increase in Cu/Ni occurred, representing 60% of the total change in the record. The accelerated eutrophication eventually led to an ecosystem regime transition from an oligotrophic state in the ESE into a eutrophic lake state in 1950. This interpretation of an ecosystem regime shift is supported by the early data from phytoplankton observations of water samples from Lake Erie^36^. Moreover, onset of a eutrophic lake state coincided with the destruction of the heavy population of burrowing mayflies (*Hexagenia spp*.) in the early 1950s^37^. Finally, high values of Cu/Ni characterize the PIE. Two intervals with elevated Cu/Ni values (E1 and E2 in Figure 5a) are concurrent with the historical and contemporary episodes of massive algal blooms in 1960s and 2000s^9^. These results further attest to the prevalence of a eutrophic state in the lake over the past six decades.

The long-standing increasing trend of Cu/Ni in sediments from the Sandusky basin is also evident in the previously published trace metal sediment record from the central basin^16^ (see Figure 5b). There was an identical 0.3 increase in Cu/Ni between 1880 and 1950, representing a comparable change in the lake's biological productivity occurred in the central basin. This confirms our assertion that an ecosystem regime shift occurred in the lake during the CIE. Similarly, the trace metal record from the central basin shows two pronounced increases in Cu/Ni in the late 1950s and 1995, corresponding well to the two episodes of massive algal blooms. However, the central basin Cu/Ni record was more sensitive to changes in lacustrine productivity, particularly during the PIE. Relatively low values of Cu/Ni characterize a period between 1970 and 1990, representing a major improvement in the pelagic waters of the lake, a conclusion in agreement with a phytoplankton biomass reduction observed from field collections^12^. However, the concurrent changes in Cu/Ni as recorded in the Sandusky basin were quite minimal, suggesting that the water quality improvement from early rigorous abatement efforts was limited within the lake's pelagic waters. This explains why algal species typical of eutrophic conditions still dominated while a significant biomass reduction was observed from 1970 through 1983–87^12^,^38^.

The use of Cu/Ni as a proxy for lacustrine productivity is supported by independent isotope data from Lake Erie. A sediment organic matter δ^13^C record from the eastern basin^15^ exhibits a paralleled long-term increasing trend between 1880 and 1950 and a subsequent peak in 1960 (Figure 5c). Increases in δ^13^C of organic matter can be interpreted to indicate increases in the lake's biological productivity and *vice versa*^39^. The carbon isotopic record is consistent with the Cu/Ni records from the Sandusky and central basins. Particularly remarkable is the large long-lasting increasing trend of lacustrine productivity as shared by the elemental and isotopic data from the lake's three different basins, attesting to the existence of the ecosystem regime shift caused plausibly by enhanced nutrient loading into Lake Erie^15^. Within the age uncertainty, the subsequent peak in δ^13^C essentially coincided with the historical episode of massive algal blooms in 1960s.

### Lake-level fluctuations and biogeochemical cycling

Comparison of these proxy data with the lake's instrumental lake-level record back to 1860 allows us to reveal some important aspects of corresponding changes in the lake's biological productivity and hydrological conditions (Figure 5d). For instance, the large long-standing increasing trend of lacustrine productivity was virtually mirrored by a concurrent long-term lake-level lowering trend during the CIE. The two episodes of massive algal blooms (E1 and E2), as highlighted in Figure 5d, coincided with low stands in 1960s and 2000s. These correspondences underscore the influence of climatic and hydrological changes on the nutrient dynamics, biological productivity, and distributions of trace metals in the lake. Low stands may facilitate sediment mobilization and resuspension in the shallow western basin, increase the internal biogeochemical cycling, and sustain an enhanced loading of phosphorus and other trace elements in Lake Erie. On the other hand, high stands may subdue lake sediment resuspension and reduce nutrient enrichment by dilution.

Evidence for pronounced in-lake biogeochemical cycling comes from the western basin, in which concentrations of these trace metals in suspended sediments are rather uniform (Figure 6). We argue that the absence of thermocline in the western basin allows lake-bottom sediment resuspension and thus enhances metal cycling^25^. However, the influence of metal cycling from bottom sediment resuspension may be minor in the central and eastern basins due to the presence of summer stratification. This inter-basin structural variation may contribute to some of the discrepancies between the two Cu/Ni records from the Sandusky and central basins. In contrast, there is a great deal of similarity between the isotopic and metal records from the two seasonally-stratified central and eastern basins (Figure 5b, c). Regardless of the potential influence from bottom sediment resuspension and biogeochemical metal cycling in the basin, our results from the Sandusky basin are broadly consistent with the isotopic and metal data from the central and eastern basins in indicating that the large ecosystem change occurred between 1880 and 1950. Such a large ecosystem shift was seemingly attributed to increased nutrient inputs from anthropogenic sources^15^.

**Figure 1:**
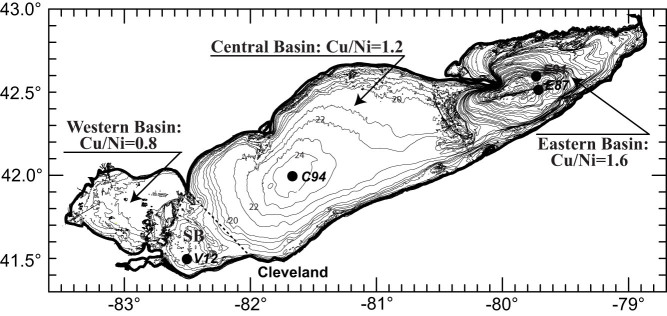
Lake Erie bathymetric map showing locations of coring sites of core V12 in the Sandusky basin, core C94^16^ in the central basin, cores E87^15^ and E91^18^ in the eastern basin. The Cu/Ni ratios are derived from suspended particulate matter trapped from the western, central and eastern basins^25^. SB: Sandusky basin. The contour data (in meters) is extracted from the NOAA National Geophysical Data Center, 1999^55^.

**Figure 2:**
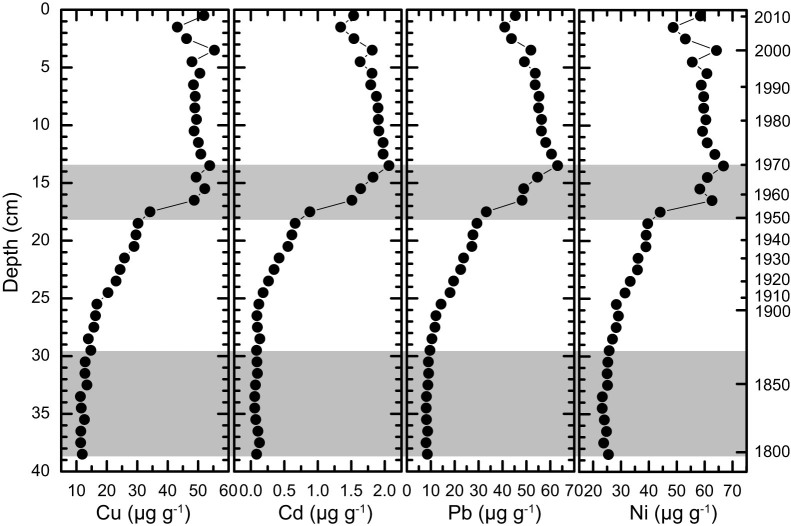
Concentration profiles of Cu, Cd, Pb, and Ni in the sediments from core V12 taken from the Sandusky basin in Lake Erie, off the Vermilion coast of northern Ohio, USA. The horizontal bars highlight intervals with distinct rates of changes in trace metal concentrations.

**Figure 3:**
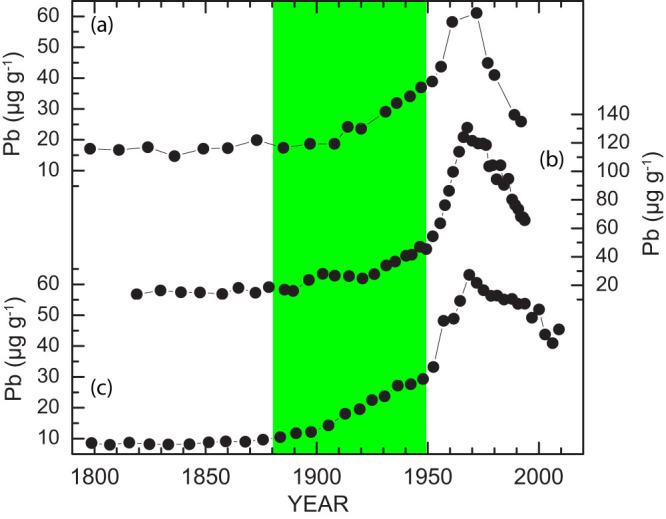
Comparison of lead concentration records derived from different sites in Lake Erie. (a) Pb data from core E91 from the eastern basin^18^. (b) Pb data from core C94 from the central basin^16^. (c) Pb data from core V12 from the Sandusky basin. The green bar highlights the coal-dominated industrial era (CIE) period between 1880 and 1950.

Changes in Cu/Ni of lake waters can be induced by variations in the anthropogenic metal emissions and in-lake metal differentiation processes. Although the amount of trace metal emissions has changed over time, variations in Cu/Ni ratio of the total anthropogenic inputs may have been negligible because the consumption ratio of Cu over Ni has changed little over the past century^40^. The significant aspects of trends and changes identified above suggest that these records are responding to changes in the lake's biological productivity. Lake Erie is fed largely by the Detroit River and other major tributaries (e.g., the Maumee and Sandusky Rivers). The majority of Cu and Ni derived from these rivers^26^ mix and interact with lake waters in the shallowest western basin where most of the lake's algal blooms occur. Lake waters flow through the central and eastern basins before reaching the Niagara Falls. It was observed that levels of Ni, Pb and Cd in suspended particulate matter decreased gradually while levels of Cu in suspended particulate matter increased progressively over the course of water movement in the lake (Figure 6). As a result, the mean ratio of Cu/Ni increased longitudinally from 0.8 in the western basin to 1.2 in the central basin to 1.6 in the eastern basin (Figure 1). Moreover, there was a similar trend of Cu/Ni in dissolved forms (Table 1), as observed in 1993 by Nriagu, et al.^26^. We attribute this longitudinal change in Cu/Ni to in-lake metal differentiation caused by a cumulative effect of the preferential phytoplankton uptake of Ni versa Cu. The two trace metals are to varying degrees organically complexed^41^. Changes in Cu/Ni in Lake Erie are most probably attributed to a stronger organic complexation of Cu which, in turn, limits its bioavailability and uptake by phytoplankton^42^,^43^.

### Pb/Cu and Cd/Pb

The Pb/Cu record appears to track well the changes in anthropogenic inputs, in particular, the Pb reduction induced by restricted use of leaded gasoline since its peak usage in 1972^44^. In fact, the rates of reduction in Pb/Cu in the lake were somewhat exaggerated due to their differentiation mechanisms. Most of the Cd and Pb are delivered via the atmosphere from various anthropogenic sources such as fossil fuel burning and non-ferrous metal extractions^26^,^45^. In contrast, most of the Cu and Ni are delivered via the Detroit River and other major rivers from municipal and industrial effluents^26^. Because of a strong complexation with organic ligands, there are relatively high levels of dissolved Cu and Ni in Lake Erie (Table 1). The calculated residence times for Cu and Ni are about 15 years, much longer than those for Cd (0.23 years) and Pb (0.02 years)^26^. This accounts for the slower reduction of Cu and Ni in the Sandusky basin.

**Figure 4:**
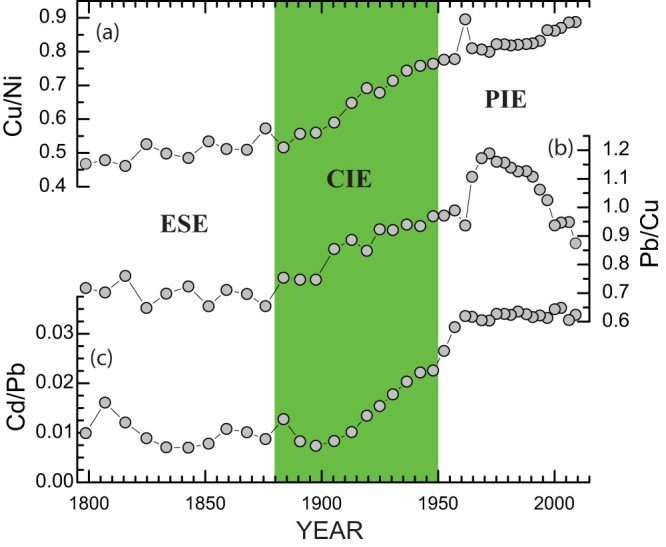
Changes in trace metal ratios of the sediments from core V12 from the Sandusky basin of Lake Erie. (a) Cu/Ni ratios. (b) Pb/Cu ratios. (c) Cd/Pb ratios. ESE: European settlement era (1800–1880); CIE: coal-dominated industrial era (1880–1950); PIE: petroleum-dominated industrial era (1950–2010). The vertical green bar highlights the CIE period, during which Cu/Ni and other metal ratios increased substantially.

**Figure 5:**
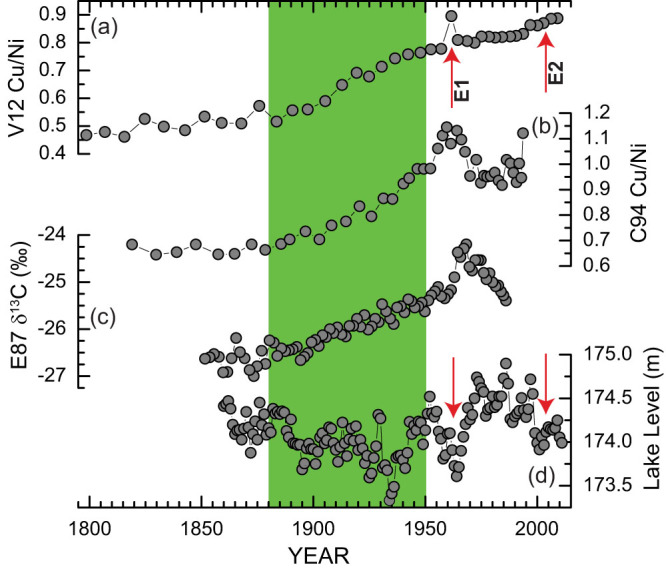
Comparison of the V12 Cu/Ni record with the C94 Cu/Ni, E87 δ^13^C, and lake-level data from Lake Erie. (a) Cu/Ni data from core V12 in the Sandusky basin. (b) Cu/Ni data from core C94 in the central basin^16^. (c) Organic matter δ^13^C data from core E87 in the eastern basin^15^. (d) Historical lake-level record of Lake Erie^54^. E1 and E2 denote the two episodes of massive algal blooms that coincided with low stands in 1960s and 2000s. The vertical green bar highlights the regime shift that occurred between 1880 and 1950.

**Figure 6:**
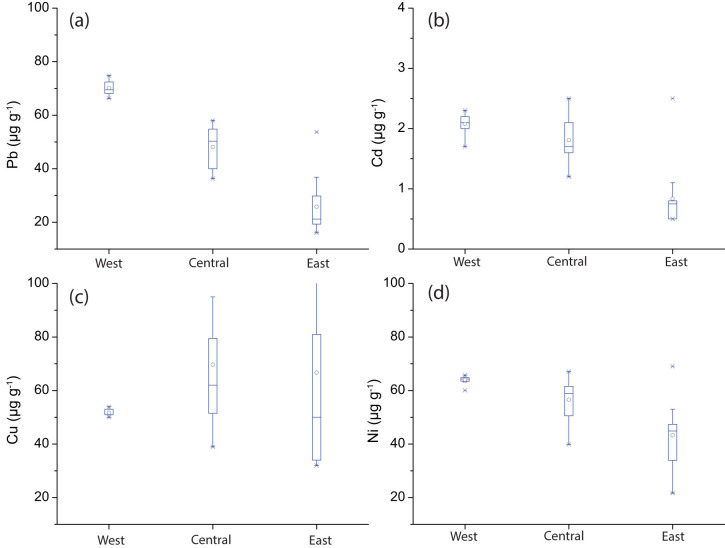
Concentrations of trace metals in suspended particulate matter trapped between 2000 and 2002 from the western, central and eastern basins of Lake Erie. (a) Pb concentrations. (b) Cd concentrations. (c) Cu concentrations. (d) Ni concentrations. Original data are from Marvin, et al.^25^.

The most intriguing feature in our sediment record is that values of Cd/Pb have remained nearly constant (0.034) since 1960 (Figure 4c), close to those of Cd/Pb in suspended and surficial sediments across the three basins (Table 1). More importantly, the Cd/Pb transition from a rising mode to a constant mode was fairly unusual as it coincided with the first episode (E1) of massive algal blooms in the lake. This strongly suggests that a large shift in the lake's trophic state and biological productivity has triggered a significant change in the distribution of these trace metals. We argue that certain biologically-produced organic ligands from this eutrophic lake^46^ may have facilitated the binding of dissolved Cd and thus reduce the activity of free Cd^2+^. Most of the Cd in natural waters is in complex forms^47^. It has been showed that the ratio of free Cd^2+^ over the total dissolved Cd typically ranged from 1–3% in eutrophic lakes to 5–9% in rivers^45^,^47^,^48^. Additionally, observational data from Lake Erie show that the ratio of dissolved Cd/Pb was at least one order greater than that of suspended and surficial sediments (Table 1). These results further support our assertion that a large change in the trophic state and biological productivity has significantly enhanced the biogeochemical differentiation processes in the lake over the last half century.

Our results provided some novel perspectives on the ecosystem changes in terms of the lake's biological productivity over the past two centuries. We found multiple lines of evidence that Lake Erie experienced a marked long-term ecosystem transition between 1880 and 1950, due largely to an increased nutrient loading from anthropogenic sources. Lake Erie transitioned from an oligotrophic state prior to 1880 to a eutrophic lake state after 1950. The lake's biological productivity has ever since remained fairly high, despite the fact that rigorous nutrient abatement programs began as early as in 1969 and that a target reduction of tributary loading was reached in 1983^49^,^50^,^51^. Our results also showed that the anthropogenically-induced eutrophication history of Lake Erie was compounded by regional climate change. Additionally, this work underscored the role of in-lake biogeochemical cycling in sustaining the loadings of dissolved trace elements in the lake. Further efforts to quantify the contributions to the trace metal and other nutrient loadings by the in-lake biogeochemical cycling are essential for better management practices in this already eutrophic lake.

## Methods

### Sediment coring and subsampling

Core V12 (41.500°N, 82.467°W; in 13 m water depth) was recovered from the Sandusky basin with an HTH sediment gravity corer, transported onshore immediately, extruded vertically, and subsampled at every 1 cm interval. A total of 39 sediment samples were oven dried at 60°C and homogenized with a mortar and pestle.

### Sample digestion

A microwave-assisted acid digestion procedure adapted from the US EPA method 3015A was used for sample preparation. Approximately 0.1 g of dried sediments was loaded into Teflon vessels with 8 ml 16 M HNO_3_ and 2 ml 12 M HCl. The vessels were capped and placed into a microwave that was programed to heat samples to temperatures of 160°C ± 4°C in 15 minutes and maintain the temperatures between 165–170°C for another 10 minutes. The digested samples were air cooled back to room temperature, transferred to polypropylene sample tubes, diluted to 50 ml with reagent water, and then gravity filtered.

### Elemental analysis

The filtered solutions were analyzed with a Thermo Scientific iCAP 6000 Series ICP optical emission spectrometer equipped with a CETAC ASX-520 autosampler. A total of 22 elements were measured simultaneously but only the results of the four trace metals were studied and presented in this report. Spike-and-recovery experiments were conducted on selected samples, with the average recovery rates ranging from 84 to 90%. The relative standard deviations of eight continuing calibration verification (CCV) standards were 0.8% for Ni, 0.9% for Cd, 1.7% for Pb, and 4.0% for Cu.

### Radiometric dating

**Figure 7:**
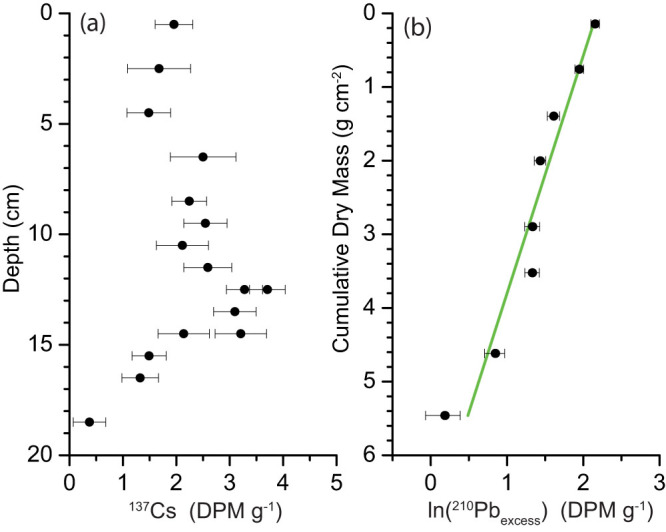
Profiles of nuclide activities in the topmost section of core V12 from the Sandusky basin of Lake Erie. (a) Cs-137 activity. (b) Unsupported Pb-210 activity. The linear regression line (in green): y = 7.03275 − 3.229766 × ln(^210^Pb_excess_), *r* = −0.97. The sedimentation rate estimated: (−3.229766)(0.6931)/(−22.3) = 0.101 (g cm^−2^ yr^−1^).

Selected sediment samples from core V12 were measured on Pb-210, Cs-137, and Ra-226 at Flett Research Laboratory in Canada. The Ra-226 activities measured at four depths indicate that the background levels of Pb-210 activity vary slightly with depth in the core. The Cs-137 activity peaks at a depth between 14 and 15 cm (Figure 7a), indicating the 1963 maximum in Cs-137 atmospheric fallout^52^. The unsupported Pb-210 activity estimated decreases with depth nearly exponentially (Figure 7b).

### Core chronology

We applied a linear regression model to deduce the average sedimentation rate^53^. As shown in Figure 7b, the deduced rate of sedimentation is 0.101 g cm^−2^ yr^−1^. Alternatively, we took the Pb peak that was induced by the peak use of leaded gasoline in 1972^44^ as an independent age marker. The rate of sedimentation estimated is a 0.097 g cm^−2^ yr^−1^, which is not statistically different from that obtained from the regression model. Lastly, the deduced age control is also in agreement with the age marker from Cs-137 data (Figure 7a).

## Author Contributions

F.Y. and R.D. conceived the study and collected the sediment core. R.D. and C.S. performed the ICP-OES analyses. F.Y. conducted the data analysis, prepared all the figures, and wrote the manuscript. All authors contributed to interpreting the results and editing the manuscript.

## Figures and Tables

**Table 1:** Concentrations and ratios of trace metals in dissolved forms and suspended, surficial, and cored sediments of Lake Erie^a^

	Period	Cu	Ni	Pb	Cd	Cu/Ni	Pb/Cu	Cd/Pb
	*Dissolved forms*							
Eastern Basin^b^	1993	933 ± 27	724 ± 98	5.0 ± 2.8	2.4 ± 1.2	1.31 ± 0.19	0.005 ± 0.003	0.671 ± 0.535
Central Basin^b^	1993	858 ± 110	947 ± 237	6.4 ± 7.1	3.2 ± 2.3	0.96 ± 0.24	0.007 ± 0.007	0.697 ± 0.575
Western Basin^b^	1993	765 ± 47	871 ± 102	8.4 ± 1.6	3.2 ± 2.0	0.89 ± 0.12	0.011 ± 0.002	0.294 ± 0.209
	*Suspended sediments*							
Eastern Basin^c^	2001–2002	66.7 ± 44.4	43.3 ± 9.6	25.8 ± 8.7	0.84 ± 0.46	1.67 ± 1.24	0.541 ± 0.310	0.033 ± 0.015
Center Basin^c^	2001–2002	69.7 ± 28.6	56.6 ± 8.2	48.1 ± 7.8	1.81 ± 0.35	1.24 ± 0.47	0.763 ± 0.254	0.038 ± 0.010
Western Basin^c^	2001–2002	51.7 ± 1.6	64.0 ± 1.7	70.1 ± 3.0	2.08 ± 0.21	0.81 ± 0.03	1.358 ± 0.069	0.030 ± 0.002
	*Surficial sediments*							
Eastern Basin^d^	1997–1998	32.4 ± 8.5	37.7 ± 11.1	30.8 ± 12.7	0.96 ± 0.17	0.82 ± 0.15	0.86 ± 0.43	0.040 ± 0.029
Central Basin^d^	1997–1998	42.6 ± 12.5	45.6 ± 11.7	51.2 ± 19.1	1.68 ± 0.83	0.89 ± 0.22	1.27 ± 0.44	0.039 ± 0.031
Western Basin^d^	1997–1998	43.6 ± 11.3	43.6 ± 12.7	47.1 ± 16.8	1.53 ± 0.97	0.99 ± 0.14	1.10 ± 0.21	0.034 ± 0.017
Western Basin^e^	2007–2008	27.3 ± 13.5	28.3 ± 15.0	75.5 ± 36.3	2.84 ± 1.18	0.98 ± 0.25	2.96 ± 0.72	0.041 ± 0.010
	*Cored sediments*							
Sandusky Basin^f^	1800–1880	12.4 ± 1.1	25.5 ± 1.0	8.6 ± 0.5	0.08 ± 0.02	0.50 ± 0.03	0.70 ± 0.04	0.010 ± 0.003
Sandusky Basin^f^	1880–1950	23.3 ± 6.7	34.2 ± 5.5	20.8 ± 7.6	0.36 ± 0.26	0.67 ± 0.09	0.87 ± 0.08	0.015 ± 0.006
Sandusky Basin^f^	1950–2010	49.7 ± 2.8	59.5 ± 4.2	52.6 ± 5.9	1.77 ± 0.20	0.84 ± 0.03	1.06 ± 0.10	0.034 ± 0.001

^a^Units are in μg/g except dissolved trace metals (in ng/L).

^b^Nriagu et al. (1995).

^c^Marvin et al. (2007).

^d^Painter et al. (2001).

^e^Opfer et al. (2011).

^f^This study.
